# Marriage among New York Plutocrats

**Published:** 1891-10

**Authors:** Edgar Fawcett


### MARRIAGE AMONG NEW YORK PLUTOCRATS.

Marriage among the New York snobs and plutocrats, ordinarily treats human affection as though it were a trifling optic malady to be cured by a few drops of corrective lotion. Daughters are trained by their mothers to leave no efforts untried, short of those absolutely immoral, in winning wealthy husbands. Usually the daughters are tractable enough. Rebellion is rare with them; why should it not be? Almost from infancy (unless when their parents have made fortunes with prodigious quickness) they are taught that matrimony is a mere hard bargain, to be driven shrewdly and in a spirit of the coolest mercantile craft. Sometimes they do really rebel, however, mastered by pure nature, in one of those tiresome moods where she shows the insolence of defying bloodless convention. Yet nearly always capitulation follows. And then what follows later on? Perhaps heart-broken resignation, perhaps masked adultery, perhaps the degradation of public divorce. But usually it is no worse than a silent disgusted slavery, for the American woman is notoriously cold in all sense of passion. and when reared to respect "society," she is a snob to the core. Some commentators aver that it is the climate which makes her so pulseless and prudent. This is possible. But one deeply familiar with the glacial theories of the fashionable New York mother might find an explanation no less frigid than comprehensive for all her traits of acquiescence and decorum. How many of these fashionable mothers ask more than a single question of the bridegrooms they desire for their daughters? That one question is simply: "What amount of money do you control?" But constantly this kind of interrogation is needless. A male "match" and "catch" finds that his income is known to the last dollar long before he has been graduated from the senior class at Columbia or Harvard. Society, like a genial feminine Briaræus, opens to him its myriad rosy and dimpled arms. He has only to let a certain selected pair of these clutch him tight, if he is rich enough to make his personality a luring prize. Often his morals are unsavory, but these prove no impediment. The great point with plutocracy and snobbery is to perpetuate themselves—to go on producing scions who will uphold for them future generations of selfishness and arrogance. One sees the same sort of procreative tendency in certain of our hardiest and coarsest weeds. Sometimes a gardener comes along with hoe, spade, and a strong uprooting animus. In human life that kind of gardener goes by the ugly name of Revolution. But we are dealing with neither parables nor allegories. Those are for the modish clergymen of the select and exclusive churches, and are administered in the form of dainty little religious pills which these gentlemen have great art in knowing how to palatably sugar.—EDGAR FAWCETT, in *Arena.*

